# Niraparib for the treatment of metastatic ccRCC in a patient with *CDK12* and *RAD51C* mutations: a case report

**DOI:** 10.3389/fphar.2024.1396606

**Published:** 2024-06-17

**Authors:** Xiaolong Yue, Chenkang Yang, Dandan Cao, Yue Li

**Affiliations:** ^1^ Medical Oncology Department, Affiliated Tumor Hospital, Harbin Medical University, Harbin, China; ^2^ Genetron Health (Beijing) Co., Ltd., Beijing, China

**Keywords:** ccRCC, PARPi, CDK12, RAD51C, niraparib

## Abstract

**Background:**

Niraparib, a poly ADP-ribose polymerase inhibitors (PARPi), has been widely applied in the intervention of epithelial ovarian, fallopian tube, or primary peritoneal cancer. Nevertheless, as of the present moment, there are limited instances demonstrating favorable outcomes stemming from niraparib therapy in patients with clear cell renal cell carcinoma (ccRCC).

**Case presentation:**

Here, we report a case of a 50-year-old patient with ccRCC who subsequently developed distant metastasis. The patient received monotherapy with pazopanib and combination therapy with axitinib and tislelizumab, demonstrating limited efficacy. Liquid biopsy revealed missense mutations in the *CDK12* and *RAD51C* of the homologous recombination repair (HRR) pathway, suggesting potential sensitivity to PARPi. Following niraparib treatment, the patient’s condition improved, with no significant side effects.

**Conclusion:**

In summary, patients with ccRCC harboring HRR pathway gene mutation may potentially benefit from niraparib. This will present more options for ccRCC patients with limited response to conventional treatments.

## Introduction

Renal cell carcinoma (RCC) constitutes 90%–95% of kidney cancers in adults (Siegel et al., 2018), encompassing clear cell RCC (ccRCC), papillary RCC (pRCC), chromophobe RCC (chRCC), and other rare subtypes (Hsieh et al., 2017). Among these, ccRCC is the most common subtype, accounting for about 75% of cases (Li et al., 2019). It originates from renal tubule epithelial cells and is primarily managed through surgical intervention. However, even after successful surgery, approximately 30% of patients may experience postoperative metastasis (Hsieh et al., 2017). The standard of care for metastatic ccRCC comprise anti-angiogenic agents, mammalian target of rapamycin (mTOR) inhibitors, et al. (Yang et al., 2023). While a significant number of individuals derive relief from standard treatment, there are cases where some individuals fail to derive apparent benefits or eventually develop resistance to the standard interventions (Posadas et al., 2013).

The U.S. Food and Drug Administration (FDA) has approved four poly ADP-ribose polymerase inhibitors (PARPi): olaparib, niraparib, talazoparib, and rucaparib (Lai et al., 2022). In alignment with other PARPi, niraparib is an innovative cancer treatment based on the synthetic lethality effect (Lai et al., 2022). Niraparib has been widely applied in the intervention of epithelial ovarian, fallopian tube, or primary peritoneal cancer. Recent literature suggests that the applicability of PARPi, including niraparib, may extend to a broader range (Eiriz et al., 2023). Niraparib elevated the objective response rate (ORR) among ovarian cancer patients with HRD-positive tumors, regardless of the presence of BRCA mutations (Eiriz et al., 2023). On 11 August 2023, the FDA approved the combined use of niraparib and abiraterone acetate for the treatment of castration-resistant prostate cancer (mCRPC) in adult patients with deleterious or suspected deleterious BRCA mutations. Simultaneously, for other PARPi, on 30 June 2023, the FDA expanded approval for talazoparib to include the care of prostate cancer with mutations in multiple HRR pathway genes, such as *BRCA1*, *BRCA2*, *PALB2*, *ATM*, *ATR*, *CHEK2*, *FANCA*, *RAD51C*, *NBN*, *MLH1*, *MRE11A*, and *CDK12* (Akbıyık and Ürün, 2023).

Olaparib, an earlier-generation PARPi compared to niraparib, is presently under assessment as a monotherapy in a Phase II clinical trial for patients with DDR-altered RCC, with an anticipated completion date in 2025 (NCT03786796). Currently, there is insufficient clinical evidence to support the efficacy of PARPi for ccRCC patients with HRD. Here, we report for the first time a case of a ccRCC patient with *RAD51C* and *CDK12* mutations benefiting from niraparib treatment.

## Case description

**FIGURE 1:**
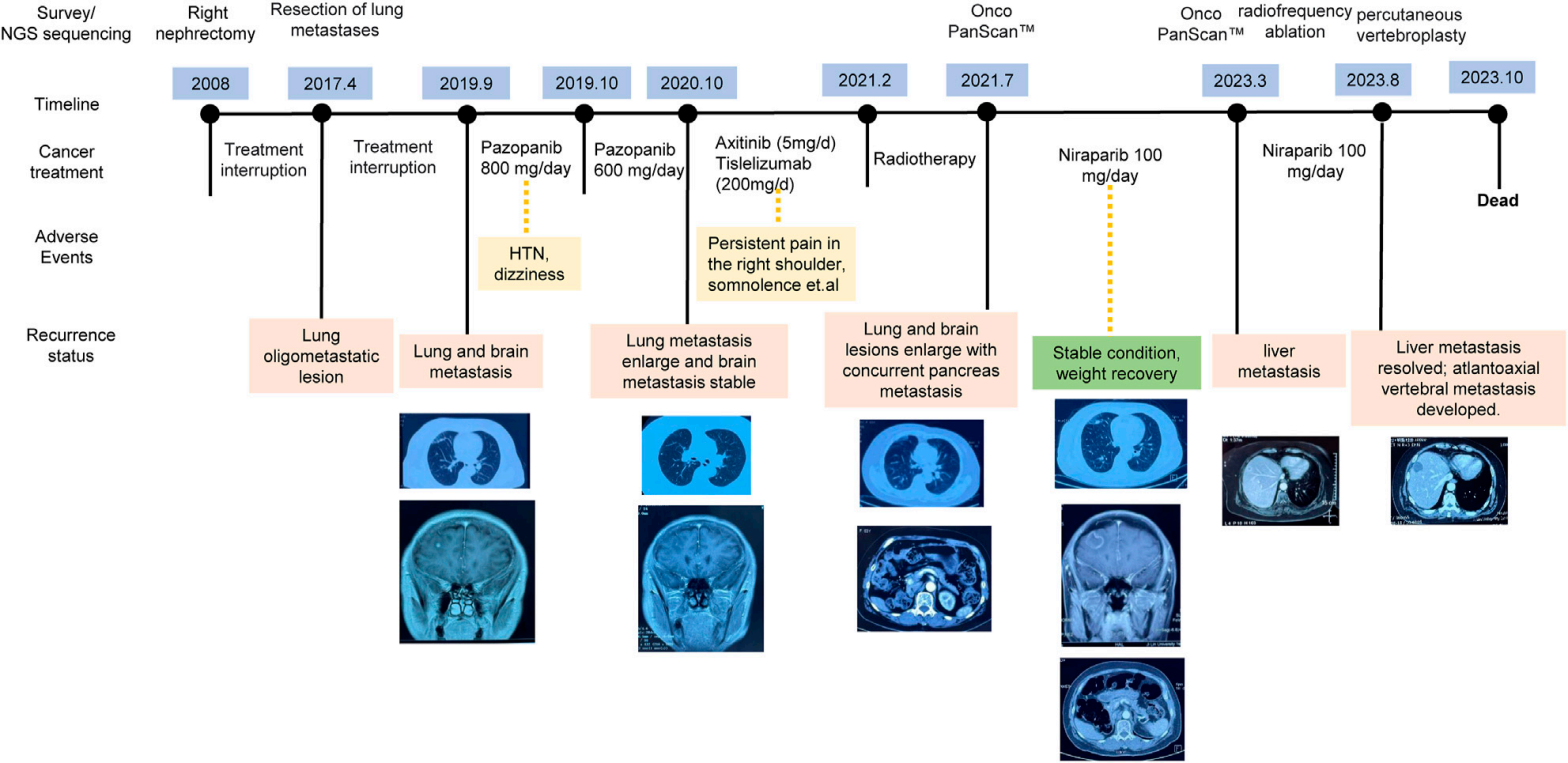
Overview of Treatment Interventions in a 50-Year-Old Patient Diagnosed with ccRCC.

In this case study, we outline the treatment course of a 50-year-old patient diagnosed with ccRCC and a history of diabetes mellitus, as depicted in Figure 1. In 2008, the patient was diagnosed with stage II ccRCC in the right kidney and subsequently underwent a nephrectomy. After the initial surgery, the patient abstained from further medical interventions until the identification of lung metastases in 2017. Subsequent surgery addressed recurrent lung metastatic lesions, without other interventions. Postoperative immunohistochemistry revealed expression of CD10, PAX-8, and Vimentin, aligning with the patient’s medical history, indicating metastatic ccRCC.

In September 2019, lung and brain metastases were identified, prompting adherence to the treatment guidelines outlined by the National Comprehensive Cancer Network (NCCN) (Jonasch, 2019). The patient initiated first-line pazopanib therapy at an initial oral dose of 800 mg daily. However, after 1 month, the patient presented elevated blood pressure and dizziness, resulting in a reduction of the pazopanib dosage to a reduced oral intake of 600 mg per day. A year later, the patient presented with increased somnolence, confusion, persistent headaches, and the CT image revealed larger lesions in the lung and brain. Brain edema and two nodular shadows in the right frontal lobe were also identified through imaging. Following NCCN guidelines, the patient initiated to a treatment consisting of axitnib (5 mg/day) combined with tislelizumab (200 mg/day). During the treatment, the patient developed persistent right shoulder pain.

In February 2021, the patient presented with severe headaches, profound somnolence, substantial weight loss, and impaired consciousness. While radiotherapy provided partial relief from symptoms, by July 2021, there occurred worsening lung and brain metastases, as well as concurrent pancreatic metastasis. Comprehensive genomic profiling (Onco PanScan™) was conducted, revealing two import gene mutations: the missense mutation in *CDK12* and *RAD51C*. Considering the limited effectiveness with conventional treatments for the patient and the identification of the gene mutations in the HRR pathway, we recommend PARPi therapy. After discussing with her family, the patient provided informed consent to participate in an individualized treatment with niraparib at a daily dosage of 200 mg. One week later, a blood test revealed thrombocytopenia, so the dosage was reduced to 100 mg per day.

After taking 100 mg niraparib per day, the patient exhibited a substantial alleviation of headaches without observable adverse effects. The lesions remained stable, and her overall health improved, including notable weight gain. Over the ensuing 2 years, routine check-ups conducted every 3 months revealed no signs of metastasis and normal blood test findings. In March 2023, liver imaging indicated the presence of metastasis, prompting liver radiofrequency ablation. Subsequent Onco PanScan™ testing in March failed to detect *RAD51C* and *CDK12* mutations, suggesting the potential development of drug resistance. However, niraparib treatment was not discontinued (Figure 1). In August of the same year, she experienced head and neck pain due to atlantoaxial vertebral metastasis. She underwent percutaneous vertebroplasty, which caused occlusion of pulmonary vasculature, leading to a deterioration in her condition with an ECOG score of 4. She passed away in October.

## Discussion

The current standard treatment for metastatic ccRCC encompass anti-angiogenic agents, mTOR inhibitors, and immunotherapeutic drugs (Yang et al., 2023). After the occurrence of distant metastasis for the patient, we initially employed pazopanib and a combination of axitinib and tislelizumab for treatment. Unfortunately, the efficacy of these approaches proved to be limited, accompanied by significant side effects. Consequently, we found it necessary to explore novel treatment strategies.

**FIGURE 2:**
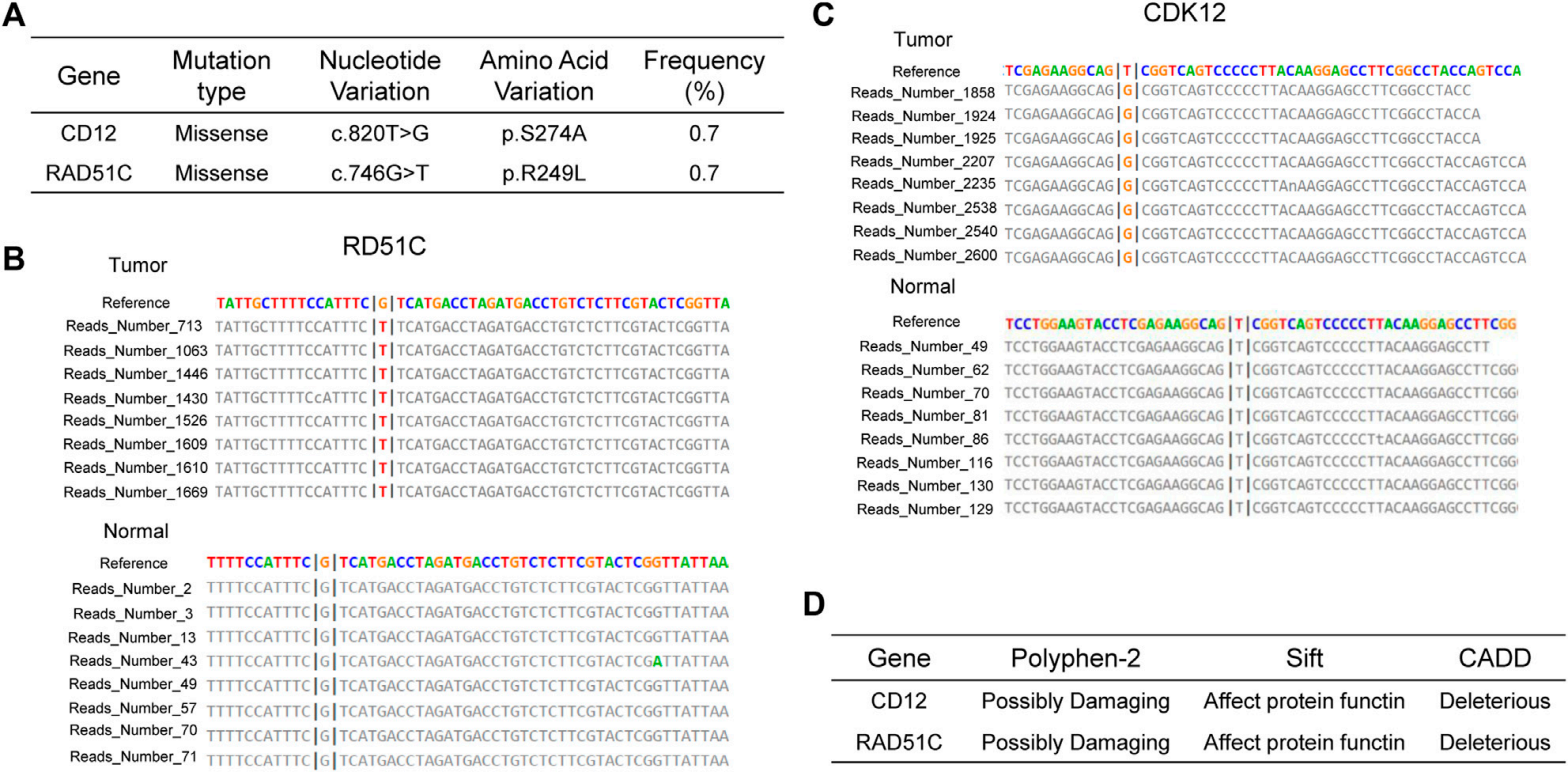
Mutational Characteristics **(A–C)** and Predicted Pathogenicity **(D)** of HRR Genes in the Patient. **(A)** Mutation sites and mutation types of *CDK12* and *RAD51C*. Mutation reads for *RAD51C* **(B)** and *CDK12* **(C)** in the tumor sample and the wild-type reads from the paired normal sample. **(D)** Prediction of functional effects of *RAD51C* and *CDK12* mutation sites by three algorithms.

In contemporary contexts, PARPi have brought about a substantial transformation in the therapeutic landscape concerning platinum-sensitive ovarian and breast cancers (Marchetti et al., 2012; Robson et al., 2017). As early as 2014, the FDA approved the utilization of PARPi for treating breast cancer in individuals with *BRCA1/2* germline mutations (Kim et al., 2015). Historically, the effectiveness of PARPi in urological cancers was believed to be constrained due to the infrequent occurrence of *BRCA* germline mutation (Pletcher et al., 2021). Hence, there is a scarcity of research on the utilization of PARPi for ccRCC. Despite this, existing studies suggest PARPi potential effectiveness for ccRCC, especially in cases where HRD are present, as demonstrated by experiments conducted on cell lines (Pletcher et al., 2021). At the same time, recent studies propose that the effectiveness of PARPi is not solely dependent on *BRCA* deficiency but may also be affected by mutations in other genes within HRR pathway (Boussios et al., 2020). In a clinical trial of prostate cancer focusing on alterations in the HRR genes, including *BRCA1*, *BRCA2*, *PALB2*, *ATM*, *ATR*, *CHEK2*, *FANCA*, *RAD51C*, *NBN*, *MLH1*, *MRE11A*, and *CDK12*, the group treated with talazoparib showed a significant improvement in progression-free survival compared to the placebo group (Fizazi et al., 2023). Soon afterwards, the FDA granted further approval for the application of talazoparib in the treatment of prostate cancer patients exhibiting mutations in HRR pathway genes, encompassing *CDK12* and *RAD51C* (Akbıyık and Ürün, 2023). Our analysis identified ccRCC patients with co-mutations in the *CDK12* and *RAD51C* genes (Figure 2A), supported by multiple reads for these mutations (Figure 2B, C). Harmfulness analysis of these mutations was conducted, and various algorithm confirmed that there are deleterious variations (Figure 2D). We propose that mutations in *CDK12* and *RAD51C* may impair the functionality of the HRR pathway, causing HRD in the patient and rendering them sensitive to PARPi. After niraparib administration, multiple lesions stabilized and the status of her remains favorable (Figure 1).

*CDK12* selectively regulates the expression of DDR pathway genes by regulating transcription elongation, consequently causing HRD. Earlier research believed the heightened susceptibility of DDR genes to *CDK12* loss, potentially attributable to their extended sequences and decreased ratios of U1 snRNP binding to intronic polyadenylation sites (Krajewska et al., 2019). Regarding *RAD51C*, as established previously, *RAD51C* deficiency leads to impaired RAD51-focus formation, and cell lines with diminished *RAD51C* expression demonstrate increased susceptibility to PARPi (Min et al., 2013). Hence, we speculate that the manifestation of HRD in patients with dual mutations in *CDK12* and *RAD51C* is attributed to alterations in both transcription elongation and RAD51-focus formation. We identified 6 patients of co-mutations in the *CDK12* and *RAD51C* genes across various cancer types among 67,083 cancer patients in the cBioPortal database (Table 1). We anticipate that future clinical trials of this co-mutations will be conducted to benefit a broader patient population. Our study contributes by presenting a ccRCC patient with HRD benefiting from the use of PARPi, adding to the growing body of evidence supporting the versatility of PARPi across various cancer types.

**TABLE 1:** Patient information with deleterious co-mutations in *CDK12* and *RAD51C*.

Sample ID	Cancer type	Amino acid variation	
		*CDK12*	*RAD51C*
TCGA-ZF-A9R7-01	Bladder Urothelial Carcinoma	P707S	R249C
P-8178	Colorectal Adenocarcinoma	R983Q	C176Y
TCGA-W3-AA1V-06	Cutaneous Melanoma	P1005L	R249C
P-0004688-T01-IM5	Upper Tract Urothelial Carcinoma	R981C	E89K
TCGA-AP-A059-01	Uterine Endometrioid Carcinoma	P653H	A279D
TCGA-A5-A0G2-01	Uterine Serous Carcinoma	E928K	R260Q

To date, there are also reported instances of employing PARPi in the management of renal cancer patients exhibiting defects in the HRR pathway. A particularly noteworthy instance is illustrated in the case study conducted by Olson et al. (Olson et al., 2016), where a patient diagnosed with pRCC and possessing an *ATM* mutation exhibited positive responses to PARPi. Additionally, Lian et al. reported the first case of a ccRCC patient benefiting from PARPi with a *BAP1* frame shift mutation (Lian et al., 2022). Similar to the case reported in this article, the patient with a *BAP1* frame shift mutation also developed lung and brain metastases. After starting niraparib treatment, the patient showed a partial response in the lungs within 2 months. The intracranial lesion also shrank due to radiotherapy, and the headache was completely relieved. This patient developed new lesions in the lungs and brain 5 months after treatment. In contrast, the patient reported in this article, who had dual *CDK12* and *RAD51C* co-mutations, did not develop new metastases during approximately 15 months of treatment. Furthermore, the case of co-mutations in the HRR pathway genes *ATR* and *BRCA2* has been reported in patients with ccRCC. However, the patient with HRD has not received PARPi therapy (Yang et al., 2021). This study presents, for the first time, the therapeutic effects of PARPi in ccRCC with mutations in genes associated with the mutation of *RAD51C* and *CDK12*. Particularly noteworthy are cases harboring mutations in *RAD51C* and *CDK12* genes, where the effectiveness of PARPi treatment has already gained FDA approval in prostate cancer.

In summary, for ccRCC patients with gene mutation in the HRR pathway, particularly in *CDK12* and *RAD51C*, PARPi demonstrate favorable therapeutic efficacy. In China, a notable number of ccRCC patients exhibit gene mutations in the HRR pathway (Chen et al., 2022), offering novel insights into drug treatment possibilities for individuals with ccRCC.

## Conclusion

In summary, patients with ccRCC harboring HRR pathway gene mutation may potentially benefit from niraparib. This will present more options for ccRCC patients with limited response to conventional treatments.

## Data Availability

The original contributions presented in the study are included in the article; further inquiries can be directed to the corresponding authors.

## References

[B1] Akbıyık I. Ürün Y. (2023). Determining magnitude of benefit from poly (ADP-ribose) polymerase inhibitors in prostate cancer. Future Oncol. 19, 2585–2591. 10.2217/fon-2023-0550 38073492

[B2] Boussios S. Karihtala P. Moschetta M. Abson C. Karathanasi A. Zakynthinakis-Kyriakou N. (2020). Veliparib in ovarian cancer: a new synthetically lethal therapeutic approach. Investig. new drugs 38, 181–193. 10.1007/s10637-019-00867-4 31650446

[B3] Chen P. Zhang Y. Bi X. Niu Y. Shayiti F. Yuan S. (2022). Abstract 5690: the landscape of homologous recombination repair gene mutations and prognosis in Chinese patients with clear cell renal cell carcinoma. Cancer Res. 82, 5690. 10.1158/1538-7445.am2022-5690

[B4] Eiriz I. Batista M. V. Freitas A. R. Martins T. Machado C. Braga S. (2023). PARP inhibitors in HRD BRCAness breast cancer patients. J. Cancer 4, 17–28. 10.46439/cancerbiology.4.049

[B5] Fizazi K. Azad A. A. Matsubara N. Carles J. Fay A. P. De Giorgi U. (2023). First-line talazoparib with enzalutamide in HRR-deficient metastatic castration-resistant prostate cancer: the phase 3 TALAPRO-2 trial. Nat. Med. 30, 257–264. 10.1038/s41591-023-02704-x 38049622 PMC10803259

[B6] Hsieh J. J. Purdue M. P. Signoretti S. Swanton C. Albiges L. Schmidinger M. (2017). Renal cell carcinoma. Nat. Rev. Dis. Prim. 3, 17009–17019. 10.1038/nrdp.2017.9 28276433 PMC5936048

[B7] Jonasch E. (2019). NCCN guidelines updates: management of metastatic kidney cancer. J. Natl. Compr. Cancer Netw. 17, 587–589. 10.6004/jnccn.2019.5008 31117033

[B8] Kim G. Ison G. McKee A. E. Zhang H. Tang S. Gwise T. (2015). FDA approval summary: olaparib monotherapy in patients with deleterious germline BRCA-mutated advanced ovarian cancer treated with three or more lines of chemotherapy. Clin. cancer Res. 21, 4257–4261. 10.1158/1078-0432.CCR-15-0887 26187614

[B9] Krajewska M. Dries R. Grassetti A. V. Dust S. Gao Y. Huang H. (2019). CDK12 loss in cancer cells affects DNA damage response genes through premature cleavage and polyadenylation. Nat. Commun. 10, 1757. 10.1038/s41467-019-09703-y 30988284 PMC6465371

[B10] Lai Y. Li Z. Lu Z. Zheng H. Chen C. Liu C. (2022). Roles of DNA damage repair and precise targeted therapy in renal cancer. Oncol. Rep. 48, 1–12. 10.3892/or.2022.8428 PMC9608312 36263616

[B11] Li Q. K. Pavlovich C. P. Zhang H. Kinsinger C. R. Chan D. W. (2019). Challenges and opportunities in the proteomic characterization of clear cell renal cell carcinoma (ccRCC): a critical step towards the personalized care of renal cancers. Seminars cancer Biol. 55, 8–15. 10.1016/j.semcancer.2018.06.004 PMC6624650 30055950

[B12] Lian B.-J. Zhang K. Fang X.-D. Li F. Dai Z. Chen W.-Y. (2022). Clinical benefit of Niraparib to TKI/mTORi-resistance metastatic ccRCC with BAP1-frame shift mutation: case report and literature review. Front. Oncol. 12, 927250. 10.3389/fonc.2022.927250 35875073 PMC9299075

[B13] Marchetti C. Imperiale L. Gasparri M. L. Palaia I. Pignata S. Boni T. (2012). Olaparib, PARP1 inhibitor in ovarian cancer. Expert Opin. investigational drugs 21, 1575–1584. 10.1517/13543784.2012.707189 22788971

[B14] Min A. Im S.-A. Yoon Y.-K. Song S.-H. Nam H.-J. Hur H.-S. (2013). RAD51C-deficient cancer cells are highly sensitive to the PARP inhibitor olaparib. Mol. cancer Ther. 12, 865–877. 10.1158/1535-7163.MCT-12-0950 23512992

[B15] Olson D. Bhalla S. Yang X. Martone B. Kuzel T. M. (2016). Novel use of targeted therapy via PARP-inhibition in a rare form of papillary renal cell carcinoma: a case report and literature review. Clin. Genitourin. Cancer 14, e445–e448. 10.1016/j.clgc.2016.03.012 27079472

[B16] Pletcher J. P. Bhattacharjee S. Doan J. P. Wynn R. Sindhwani P. Nadiminty N. (2021). The emerging role of poly (ADP-Ribose) polymerase inhibitors as effective therapeutic agents in renal cell carcinoma. Front. Oncol. 11, 681441. 10.3389/fonc.2021.681441 34307148 PMC8300201

[B17] Posadas E. M. Limvorasak S. Sharma S. Figlin R. A. (2013). Targeting angiogenesis in renal cell carcinoma. Expert Opin. Pharmacother. 14, 2221–2236. 10.1517/14656566.2013.832202 23984807

[B18] Robson M. Im S.-A. Senkus E. Xu B. Domchek S. M. Masuda N. (2017). Olaparib for metastatic breast cancer in patients with a germline BRCA mutation. N. Engl. J. Med. 377, 523–533. 10.1056/NEJMoa1706450 28578601

[B19] Siegel R. L. Miller K. D. Jemal A. (2018). Cancer statistics, 2018. CA a cancer J. Clin. 68, 7–30. 10.3322/caac.21442 29313949

[B20] Yang J. Wang J. Liang Y. Wang J. Hsu J. Liu G. (2021). ATR and BRCA2 simultaneous mutation in a ccRCC with sarcomatoid differentiation and extensive metastases: a case report. Urology 154, 45–49. 10.1016/j.urology.2021.04.025 33961890

[B21] Yang J. Wang K. Yang Z. (2023). Treatment strategies for clear cell renal cell carcinoma: past, present and future. Front. Oncol. 13, 1133832. 10.3389/fonc.2023.1133832 37025584 PMC10070676

